# Multisite Delayed Feedback for Electrical Brain Stimulation

**DOI:** 10.3389/fphys.2018.00046

**Published:** 2018-02-01

**Authors:** Oleksandr V. Popovych, Peter A. Tass

**Affiliations:** ^1^Institute of Neuroscience and Medicine, Brain and Behaviour (INM-7), Research Centre Jülich, Jülich, Germany; ^2^Department of Neurosurgery, Stanford University, Stanford, CA, United States

**Keywords:** neuronal synchronization, delayed feedback, deep brain stimulation, desynchronization, electrical pulse stimulation, closed-loop stimulation

## Abstract

Demand-controlled deep brain stimulation (DBS) appears to be a promising approach for the treatment of Parkinson's disease (PD) as revealed by computational, pre-clinical and clinical studies. Stimulation delivery is adapted to brain activity, for example, to the amount of neuronal activity considered to be abnormal. Such a closed-loop stimulation setup might help to reduce the amount of stimulation current, thereby maintaining therapeutic efficacy. In the context of the development of stimulation techniques that aim to restore desynchronized neuronal activity on a long-term basis, specific closed-loop stimulation protocols were designed computationally. These feedback techniques, e.g., pulsatile linear delayed feedback (LDF) or pulsatile nonlinear delayed feedback (NDF), were computationally developed to counteract abnormal neuronal synchronization characteristic for PD and other neurological disorders. By design, these techniques are intrinsically demand-controlled methods, where the amplitude of the stimulation signal is reduced when the desired desynchronized regime is reached. We here introduce a novel demand-controlled stimulation method, pulsatile multisite linear delayed feedback (MLDF), by employing MLDF to modulate the pulse amplitude of high-frequency (HF) DBS, in this way aiming at a specific, MLDF-related desynchronizing impact, while maintaining safety requirements with the charge-balanced HF DBS. Previously, MLDF was computationally developed for the control of spatio-temporal synchronized patterns and cluster states in neuronal populations. Here, in a physiologically motivated model network comprising neurons from subthalamic nucleus (STN) and external globus pallidus (GPe), we compare pulsatile MLDF to pulsatile LDF for the case where the smooth feedback signals are used to modulate the amplitude of charge-balanced HF DBS and suggest a modification of pulsatile MLDF which enables a pronounced desynchronizing impact. Our results may contribute to further clinical development of closed-loop DBS techniques.

## 1. Introduction

High-frequency (HF) deep brain stimulation (DBS) is the standard therapy for medically refractory Parkinson's disease (PD), where electrical pulse trains are permanently delivered via depth electrodes at high frequencies (> 100 Hz) (Benabid et al., 1991, 2009; Kuncel and Grill, 2004; Johnson et al., 2008). The mechanism of action of HF DBS is still debated (Johnson et al., 2008; Gradinaru et al., 2009; Deniau et al., 2010). HF DBS may cause side effects by stimulation of the target as well as surrounding structures (Ferraye et al., 2008; Moreau et al., 2008; van Nuenen et al., 2008; Xie et al., 2012). It is, hence, desirable to reduce the integral current delivered. Accordingly, different types of closed-loop, demand-controlled and adaptive DBS (aDBS) have been developed in computational and engineering studies (Tass, 2001, 2003; Rosenblum and Pikovsky, 2004a,b; Hauptmann et al., 2005a,b; Popovych et al., 2005, 2006; Kiss et al., 2007; Pyragas et al., 2007; Tukhlina et al., 2007; Luo et al., 2009; Popovych and Tass, 2010; Montaseri et al., 2013). Closed-loop aDBS approach received recent development in pre-clinical and clinical studies (Graupe et al., 2010; Rosin et al., 2011; Carron et al., 2013; Little et al., 2013; Priori et al., 2013; Yamamoto et al., 2013; Grahn et al., 2014; Hosain et al., 2014; Rosa et al., 2015).

Closed-loop aDBS was successfully tested in parkinsonian monkeys under acute conditions (Rosin et al., 2011). In the considered setup the globus pallidus internal (GPi) was stimulated by a short pulse train delayed by 80 ms following an action potential recorded from the primary motor cortex. Under such conditions aDBS was shown to be more effective in reducing pallidal discharge rate and pathological oscillatory neuronal activity as well as in alleviation of akinesia than the conventional continuous HF DBS (cDBS). In a proof of principle study in PD patients (Little et al., 2013), aDBS was switched on and off depending on whether the amplitude of the subthalamic nucleus (STN) local field potential (LFP) in the beta band increased above or decreased below a certain threshold. During aDBS the clinical motor scores strongly improved by about 30% better than during cDBS, whereas aDBS was switched on during 44% of the time (reduced by 56%) as compared to 100% of cDBS (Little et al., 2013). Demand-controlled aDBS was applied for suppression of essential tremor (Graupe et al., 2010). The onset of the tremor was predicted from the measured electromyographic (EMG) signal, which was used to initiate aDBS stimulation epochs. In patients with intention tremor aDBS was switched on and off based on the threshold crossing by EMG power (Yamamoto et al., 2013), where the accurately triggered switching of aDBS resulted in a complete control of the tremor.

Instead of the on-off strategy of the papers (Graupe et al., 2010; Rosin et al., 2011; Little et al., 2013; Yamamoto et al., 2013) mentioned above, the stimulation intensity can also be adapted in real time to the amplitude of the synchronized neuronal activity. Such an approach was used in a clinical study (Rosa et al., 2015), where the voltage of the stimulation was adapted to the beta-band power of the LFP each second (Rosa et al., 2015). The latter approach resembles closed-loop methods that have been developed in the past for the specifically desynchronizing control of abnormal neuronal synchronization that is characteristic for several neurological disorders including PD (Nini et al., 1995; Hammond et al., 2007), essential tremor (Schnitzler et al., 2009), epilepsy (Wong et al., 1986), and tinnitus (Llinas et al., 1999; Weisz et al., 2005; Eggermont and Tass, 2015). These techniques are feedback approaches utilizing the mean field of the synchronized population, which is measured and processed (e.g., filtered, delayed, amplified, etc.), and then fed back as stimulation signal to desynchronize neuronal populations (Rosenblum and Pikovsky, 2004a,b; Hauptmann et al., 2005a,b; Popovych et al., 2005, 2006; Kiss et al., 2007; Pyragas et al., 2007; Tukhlina et al., 2007; Luo et al., 2009; Popovych and Tass, 2010; Montaseri et al., 2013). Direct electrical stimulation of the neuronal tissue with smooth and slowly oscillating feedback signal may however cause an irreversible charge deposit in the neuronal tissue that can exceed safety limits (Harnack et al., 2004; Kuncel and Grill, 2004; Merrill et al., 2005). Two desynchronizing delayed feedback methods, linear delayed feedback (LDF) and nonlinear delayed feedback (NDF) were recently adapted and computationally tested for electrical closed-loop DBS (Popovych et al., 2017a,b). In both cases, the amplitude of charge-balanced short pulses composing the stimulation signal of the standard HF DBS was modulated by the slow feedback signal. The feedback method with such a pulsatile stimulation signal is referred to as *a pulsatile feedback stimulation* that can be used for electrical DBS.

In principle, abnormal neuronal synchronization can be counteracted in different ways. For instance, LDF (Rosenblum and Pikovsky, 2004a,b) and NDF (Popovych et al., 2005, 2006; Popovych and Tass, 2010) aim at restoring incoherent neuronal activity. In contrast, in this study we consider a multisite linear delayed feedback (MLDF) which has been designed for the control of excessive neuronal synchronization (Hauptmann et al., 2005a,b, 2007a,b; Popovych et al., 2006; Omel'chenko et al., 2008). In previous modeling studies it was observed that MLDF stimulation can counteract the synchronized dynamics by inducing clustering states, which may lead to a variety of spatio-temporal patterns of neuronal activity. Such patterns of the neuronal activity are important, for example, in the context of central pattern generators (CPG) (Marder and Calabrese, 1996; Yuste et al., 2005), where synchronized neuronal discharges have to be well-coordinated both in space and time. The MLDF stimulation approach was suggested for inducing and control of such a patterned activity, for example, in the case when physiological CPG dynamics needs to be restored (Hauptmann et al., 2007a,b; Omel'chenko et al., 2008).

We introduce a pulsatile MLDF for electrical brain stimulation and test it on a physiologically motivated model of interacting neuronal populations of STN and external globus pallidus (GPe) neurons (Terman et al., 2002; Rubin and Terman, 2004). We show that for four-site stimulation setup of MLDF with smooth stimulation signals, a weak clustering, mostly two-cluster states can be observed in a limited parameter range. For pulsatile MLDF stimulation the stimulation-induced clustering becomes even less pronounced such that the main impact of the pulsatile MLDF stimulation consists in a desynchronization, i.e., a suppression of in-phase synchronization in the stimulated population. The pulsatile MLDF stimulation is however less effective in inducing desynchronization than the pulsatile LDF. For effective desynchronization, we here introduce differential pulsatile MLDF stimulation and show this stimulation method can effectively and robustly desynchronize the model STN neurons.

## 2. Methods

### 2.1. Model

We consider a model network of STN and GPe neuronal populations suggested by Terman et al. (2002), where the dynamics of individual neurons is described by the following system:

(1) $$\begin{array}{lll} C_{m}v^{\prime } & = & -I_{\text{L}}-I_{\text{K}}-I_{\text{Na}}-I_{\text{T}}-I_{\text{Ca}}-I_{\text{AHP}}\\ -I_{\text{syn}}+I_{\text{app}}+I_{\text{stim}}, \end{array}$$

(2) $$\begin{array}{lll} {\left[\text{Ca}\right]}^{\prime } & = & \varepsilon \left(-I_{\text{Ca}}-I_{\text{T}}-k_{\text{Ca}}\left[\text{Ca}\right]\right), \end{array}$$

(3) $$\begin{array}{lll} X^{\prime } & = & \phi_{X}\left(X_{\infty }\left(v\right)-X\right)/\tau_{X}\left(v\right). \end{array}$$

In Equations (1–3), *v* is a membrane potential of the neuron, the currents *I*_L_, *I*_K_, *I*_Na_, *I*_T_, *I*_Ca_, *I*_AHP_, *I*_syn_, and *I*_app_ are the corresponding leak, potassium, sodium, low threshold calcium, high threshold calcium, afterhyperpolarization potassium, synaptic, and external current, respectively. [Ca] is the intracellular concentration of Ca^2+^ ions, and *X* = *n, h, r* are the gating variables.

The following currents are given by the same expressions for STN and GPe neurons:

$$\begin{array}{ll} I_{\text{L}}=g_{\text{L}}\left(v-v_{\text{L}}\right), & I_{\text{K}}=g_{\text{K}}n^{4}\left(v-v_{\text{K}}\right),\\ I_{\text{Na}}=g_{\text{Na}}m_{\infty }^{3}\left(v\right)h\left(v-v_{\text{Na}}\right), & I_{\text{Ca}}=g_{\text{Ca}}s_{\infty }^{2}\left(v\right)\left(v-v_{\text{Ca}}\right),\\ \begin{array}{l} I_{\text{AHP}}=g_{\text{AHP}}\left(v-v_{\text{K}}\right)\left(\left[\text{Ca}\right]/\left(\left[\text{Ca}\right]+{\text{k}}_{1}\right)\right). \end{array} \end{array}$$

On the other hand, current *I*_T_ is different for the excitatory STN neurons for the inhibitory GPe neurons:

$$\begin{array}{l} \text{STN}:I_{\text{T}}=g_{\text{T}}a_{\infty }^{3}\left(v\right)b_{\infty }^{2}\left(r\right)\left(v-v_{\text{Ca}}\right),\text{GPe}:I_{\text{T}}=g_{\text{T}}a_{\infty }^{3}\left(v\right)r\left(v-v_{\text{Ca}}\right), \end{array}$$

where *b*_∞_(*r*) = 1/(1 + exp[(*r* − θ_*b*_)/σ_*b*_]) − 1/(1 + exp[−θ_*b*_/σ_*b*_]). The functions *X*_∞_(*v*) and τ_*X*_(*v*) used in the above equations read

$$\begin{array}{l} \begin{array}{llll} X_{\infty }\left(v\right) & = & 1/\left(1+\exp\left[-\left(v-\theta_{X}\right)/\sigma_{X}\right]\right), & X=n,h,r,m,s,a;\\ \tau_{X}\left(v\right) & = & \tau_{X}^{0}+\tau_{X}^{1}/\left(1+\exp\left[-\left(v-\theta_{X}^{\tau }\right)/\sigma_{X}^{\tau }\right]\right), & X=n,h,r. \end{array} \end{array}$$

For GPe neurons τ_*r*_(*v*) = τ_*r*_ is a constant parameter.

The STN and GPe neuronal populations contain *N* = 200 neurons each and arranged on 1Dim lattices with periodic boundary conditions. Each GPe neuron receives an exitatory input from a single neighboring STN neuron and inhibits three neighboring STN neurons. The considered model was introduced to study pathological neuronal dynamics in PD and was investigated in several papers (Terman et al., 2002; Rubin and Terman, 2004; Park et al., 2011; Popovych et al., 2017a,b). The neurons are interacting via chemical synapses, where the synaptic currents are

$$\begin{array}{lll} \text{STN}:I_{\text{syn}} & = & g_{\text{G}\to S}\left(v-v_{\text{G}\to \text{S}}\right)\sum s_{\text{j}},\\ \text{GPe}:I_{\text{syn}} & = & g_{\text{S}\to G}\left(v-v_{\text{S}\to \text{G}}\right)\sum s_{\text{j}}. \end{array}$$

Summation is taken over all corresponding presynaptic neurons, where *j* is the index of neurons. The coupling strength between neurons is defined by parameters of synaptic weights *g*_S→G_ = 0.4 nS/μm^2^ (from STN to GPe) and *g*_G→S_ (from GPe to STN, will be specified below). The reversal potential for the excitatory coupling from STN to GPe *v*_S→G_ = 0 mV, and *v*_G→S_ = −100 mV for the inhibitory coupling from GPe to STN. Synaptic variables *s*_*j*_ are governed by

(4) $$\begin{array}{lll} s_{j}^{\prime } & = & \alpha H_{\infty }\left(v_{j}-\theta_{g}\right)\left(1-s_{j}\right)-\beta s_{j},\\ H_{\infty }\left(x\right) & = & 1/\left(1+\exp\left[-\left(x-\theta_{g}^{H}\right)/\sigma_{g}^{H}\right]\right). \end{array}$$

The neurons are nonidentical such that the applied currents *I*_*app*_ = *I*_app, *j*_ for STN neurons are Gaussian distributed around the mean 10 pA/μm^2^ and with the standard deviation 0.015 pA/μm^2^. For GPe neurons the parameter ε = ε_*j*_ are also Gaussian distributed around the mean 0.0055 ms^−1^ and with the standard deviation 2 · 10^−5^ ms^−1^. The other parameters for the STN and GPe neurons and their values are listed in Table S1.

### 2.2. Synchronized dynamics of STN neurons

In this study we investigate how the synchronized dynamics of the STN-GPe network can be controlled by an external stimulation. We estimate the extent of synchronization by the order parameters (Haken, 1983; Kuramoto, 1984; Tass, 1999).

(5) $$\begin{array}{l} R_{k}\left(t\right)=|N^{-1}\overset{N}{\underset{j=1}{\sum }}\exp\left(ik\psi_{j}\left(t\right)\right)|,k=1,2,\ldots , \end{array}$$

where ψ_*j*_(*t*) is the phase of neuron *j*, which attains the values ψ_*j*_(*t*_*n*_) = 2π*n*, *n* = 0, 1, … at the burst onset time moments *t*_*n*_ of the *j*th neuron. The phase linearly increases between two consecutive bursts ψ_*j*_(*t*) = 2π(*t* − *t*_*n*_)/(*t*_*n*+1_ − *t*_*n*_) + 2π*n* for *t*∈(*t*_*n*_, *t*_*n*+1_), *n* = 0, 1, … (Pikovsky et al., 2001). The order parameters *R*_*k*_(*t*) range from 0 to 1, where the values of the first order parameter R = *R*_1_ correspond to the extent of in-phase synchronization in the population. Large values of the *k*-th order parameter *R*_*k*_ together with small values of the order parameters *R*_*n*_ of smaller degree *n* < *k* are characteristic for a *k*-cluster state, where the oscillators are in-phase synchronized within the clusters, but the clusters are time (and phase) shifted with respect to each other equidistantly in the oscillation period.

**Figure 1:**
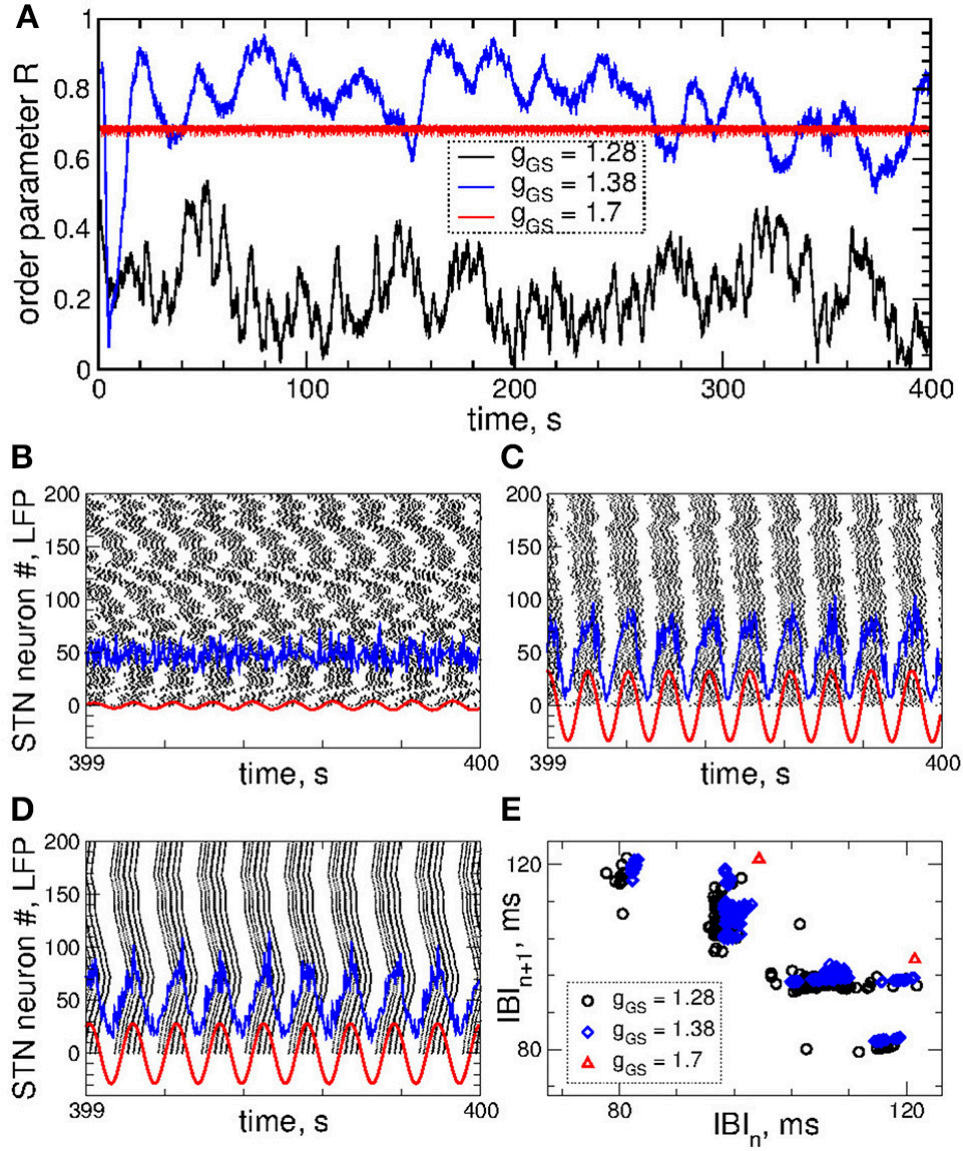
Collective dynamics of STN-GPe neurons (Equations 1–4) without stimulation. **(A)** Time courses of the order parameter *R* = *R*_1_ of the STN neurons obtained for the same initial conditions, but for different coupling parameter *g*_*G*→*S*_ as indicated in the legend. **(B–D)** The corresponding spike raster plots of *N* = 200 STN neurons, where the spike onsets are indicated by black dots for **(B)** *g*_*G*→*S*_ = 1.28 nS/μm^2^, **(C)** *g*_*G*→*S*_ = 1.38 nS/μm^2^ and **(D)** *g*_*G*→*S*_ = 1.7 nS/μm^2^. The raw LFP (ensemble-averaged synaptic variables *s*_*j*_) and filtered LFP (variable $\dot{u}$ of the linear oscillator, Equation 6) scaled by the factor 1,000 are depicted by blue and red curves, respectively. **(E)** Scatter plot of the first return map of the inter-burst intervals (IBI, time intervals between the first spikes of two consecutive bursts) of the STN neuron *j* = 100 for the values of the coupling parameter indicated in the legend.

Examples of the collective dynamics of STN neurons without stimulation (*I*_stim_ = 0 in Equation 1) are illustrated in Figure 1. Depending on the coupling strength as given by the values of parameter *g*_*G*→*S*_, STN neurons can exhibit synchronization of different extents and forms. For weak coupling, e.g., *g*_*G*→*S*_ = 1.28 nS/μm^2^, the neurons are weakly and intermittently synchronized, and the order parameter fluctuates around small values 〈*R*〉 ≈ 0.2, see Figure 1A (black curve). STN neurons exhibit desynchronized bursting dynamics (Figure 1B, black dots), where the individual bursting frequencies (number of bursts per second) of STN neurons are relatively broadly distributed in the range 9.91 ± 0.017 Hz (mean ± standard deviation), see Figure S1. The firing patters exhibit strong variation as time evolves (Figure S1). The inter-bursts intervals (IBI, time intervals between the first spikes of two consecutive bursts) vary irregularly from one burst to the next, which is illustrated in Figure 1E (black circles), where the next IBI_n+1_ are plotted vs. the previous IBI_n_ as a scatter plot of the first return map.

An increase of the coupling leads to synchronized dynamics of bursting STN neurons as illustrated by the spike raster plot in Figure 1C (black dots) for *g*_*G*→*S*_ = 1.38 nS/μm^2^. The order parameter fluctuates around a larger value 〈*R*〉 ≈ 0.8 (Figure 1A, blue curve). The individual bursting frequencies become much narrowly distributed in the range 9.75±0.003 Hz indicating that most neurons become frequency synchronized, see Figure S1. Although the firing patters still vary in time, they clearly demonstrate an in-phase synchronized dynamics of STN neurons (Figure 1C and Figure S1). The behavior of the IBIs remains however irregular (Figure 1E, blue diamonds).

Only stronger coupling can regularize the dynamics of IBIs. For example, for *g*_*G*→*S*_ = 1.7 nS/μm^2^ the IBIs can attain only two values as illustrated in Figure 1E (red triangles) such that IBIs periodically alternate between them in a period-2 manner. STN neurons synchronize at the same frequency in the very narrow range 9.06 ± 2 · 10^−6^ Hz, and the firing pattern demonstrates periodic dynamics, see Figure S1. In such a way the STN neurons become periodically synchronized for strong coupling (Figure 1D, black dots), and the order parameter is nearly constant with 〈*R*〉 ≈ 0.7 (Figure 1A, red curve).

The extent of synchronization is also reflected by the amplitude dynamics of the local field potential (LFP). The latter can be modeled as an ensemble-averaged synaptic activity of the neuronal population $LFP\left(t\right)=N^{-1}\overset{N}{\underset{j=1}{\sum }}s_{j}$ (Buzsaki, 2004), where *s*_*j*_(*t*) are the synaptic variables (Equation 4). For a more sophisticated approach see the papers (Lindén et al., 2011; Parasuram et al., 2016). The measured raw *LFP*(*t*) can be on-line filtered by means of a linear damped oscillator

(6) $$\begin{array}{l} ü+\alpha_{d}\dot{u}+\omega^{2}u=k_{\text{f}}LFP\left(t\right), \end{array}$$

where ω approximates the mean frequency of the LFP oscillations, ω = 2π/*T*, and *T* is the LFP mean period. Variable $x\left(t\right)=\dot{u}$ of Equation (6) has a zero phase shift with respect to the input raw LFP signal (Tukhlina et al., 2007), and we consider it as the filtered LFP. The other parameters of Equation (6) were chosen α_*d*_ = *k*_f_ = 0.008, which approximately preserves the amplitude of the input LFP signal (Popovych et al., 2017a,b).

The dynamics of raw and filtered LFP is illustrated in the raster plots in Figures 1B–D (blue and red curves) for the above three considered values of the coupling parameter, where large- and small-amplitude oscillations of LFP are in correspondence with strong and weak neuronal synchronization, respectively.

### 2.3. Delayed feedback stimulation

**Figure 2:**
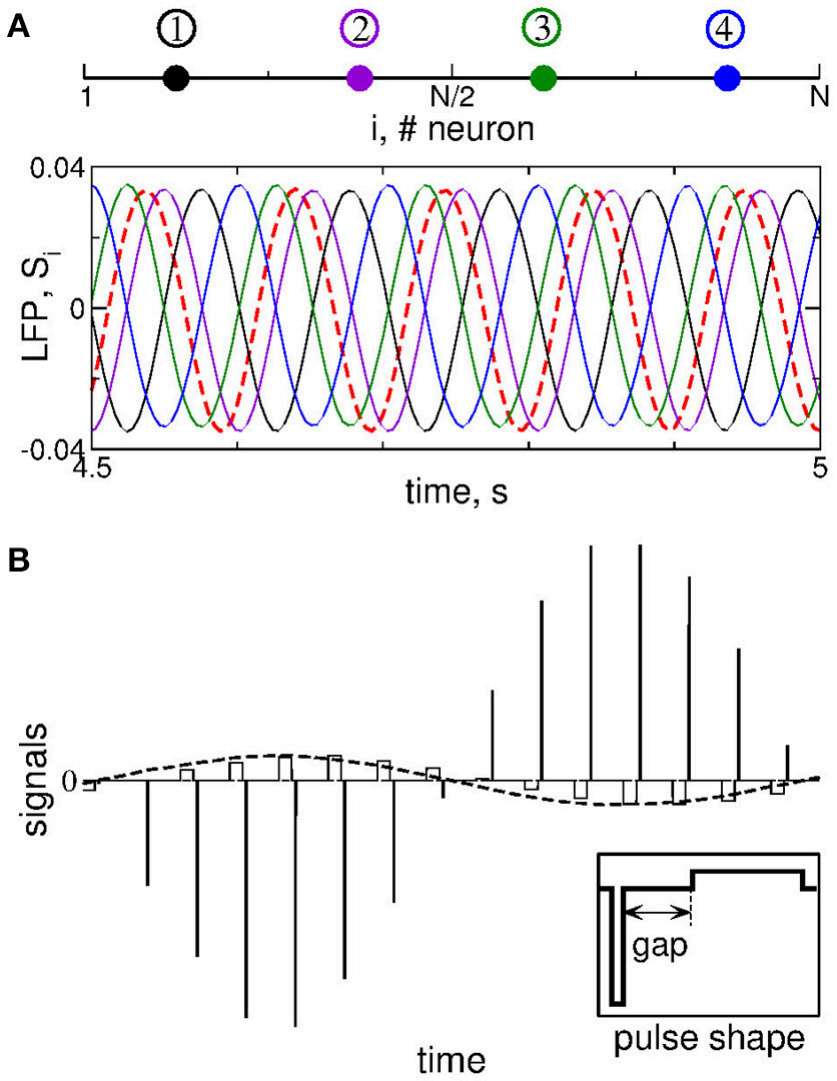
Stimulation setup of MLDF. **(A)** Four stimulation sites (color dots) equidistantly located within the 1-Dim lattice of STN neurons (upper plot) and example of the corresponding feedback signals *S*_*i*_ for delay τ = 102 ms and *K* = 1 (lower plot). The filtered LFP is depicted by the dashed red curve. **(B)** Pulsatile stimulation signal, where the amplitude of charge-balanced biphasic pulses composing a high-frequency stimulation train (solid lines) is modulated by the feedback signal (dashed curve). For details see Popovych et al. (2017a,b). The shape of a single pulse is shown in the insert, where the pulse can contain an interphase gap between the negative and positive phases of the pulse.

We stimulate the considered STN-GPe model neuronal network by multisite linear delayed feedback (MLDF), where the stimulation is administered to the STN neurons only. This stimulation techniques has been suggested and investigated in the papers (Hauptmann et al., 2005a,b, 2007a,b; Popovych et al., 2006; Omel'chenko et al., 2008). We assume that, for example, 4 stimulation sites are implanted in the STN population of *N* = 200 neurons at the equidistant lattice coordinates (index of neurons) *j* = 25, 75, 125, and 175 as schematically illustrated in Figure 2A (upper plot). The feedback stimulation signal *S*_*i*_(*t*) administered via the *i*-th stimulation site is calculated as (Hauptmann et al., 2005a,b, 2007a,b; Popovych et al., 2006; Omel'chenko et al., 2008)

(7) $$\begin{array}{l} S_{i}\left(t\right)=K\cdot x\left(t-\tau_{i}\right),\tau_{i}=\frac{11-2\left(i-1\right)}{8}\tau ,i=1,2,3,4. \end{array}$$

The delays τ_*i*_ are considered in such a form in order to achieve a time shift by *T*/4 between the feedback signals for τ = *T*. Indeed, if the measured signal *x*(*t*) is periodically oscillating with period *T*, the corresponding oscillating feedback signals (Equation 7) with neighboring indices will be time shifted with respect to each other by *T*/4 (e.g., τ_1_ − τ_2_ = *T*/4) including *S*_1_ with respect to *S*_4_, where the latter signal is considered over the next oscillation period, i.e., *T* + τ_4_ − τ_1_ = *T*/4.

To calculate the feedback signals (Equation 7), the LFP of synchronized STN neurons is measured, filtered by the linear oscillator (Equation 6), i.e., the signal $x\left(t\right)=\dot{u}$ from Equation (6) is delayed with delays τ_*i*_ from Equation (7), and amplified by the factor *K* that is a dimensionless feedback gain and will be referred to as parameter of the stimulation intensity.

Instead of four delays, we use only two of them, e.g., τ_1_ and τ_2_, and calculate the feedback signals *S*_1_ and *S*_2_ according to Equation (7). The other two feedback signals are defined by reversing polarity *S*_3_ = −*S*_1_ and *S*_4_ = −*S*_2_ (Hauptmann et al., 2005a,b, 2007a,b; Popovych et al., 2006; Omel'chenko et al., 2008). We thus have two stimulation parameters that can be varied: The stimulation intensity (feedback gain) *K* and the stimulation delay τ. We also assume that neurons within the same sub-population assigned to the corresponding stimulation site receive approximately the same signal administered via that stimulation site, which is called a segmental stimulation, see Hauptmann et al. (2005a,b), Popovych et al. (2006), Hauptmann et al. (2007a,b), and Omel'chenko et al. (2008). More precisely, the neurons *j* = 1, 2, …, 50 are stimulated with the same signal *S*_1_, neurons *j* = 51, 52, …, 100 are stimulated with the same signal *S*_2_, and so on.

The feedback signals *S*_*i*_ are illustrated in Figure 2A (lower plot) together with filtered LFP (dashed red curve). For the considered delay τ ≈ *T*, where *T* ≈ 102 ms is the mean period of LFP oscillations, the neighboring feedback signals are time shifted by approximately *T*/4 with respect to each other if the filtered LFP signal is periodic or close to that. Delayed feedback stimulation with such smooth signals may be referred to as smooth feedback stimulation. Direct electrical stimulation of the brain with such signals might violate safety requirements and cause an irreversible charge deposit to the neuronal tissue and could lead to its damage (Harnack et al., 2004; Kuncel and Grill, 2004; Merrill et al., 2005). This problem was studied recently for single-site delayed feedback stimulation (Popovych et al., 2017a,b). By a similar token, we here use a high-frequency stimulation pulse train of the standard HF DBS consisting of biphasic charge-balanced pulses (Volkmann et al., 2002; Kuncel and Grill, 2004; Butson and McIntyre, 2007), whose amplitude is modulated by the slowly oscillating feedback signals *S*_*i*_(*t*) as schematically illustrated in Figure 2B, where an example of the pulsatile stimulation current *I*_*stim*_ in Equation (1) is shown. The cathodic and anodic phases of the pulses administer the same charge of opposite polarity, and a charge-balanced stimulation is realized in this way. The resulting zero net charge injection after each short biphasic pulse can prevent from damaging nervous tissue (Lilly et al., 1955; Harnack et al., 2004; Kuncel and Grill, 2004; Merrill et al., 2005). Each pulse can contain an interphase time gap between its cathodic and anodic phases (Figure 2B, insert). We refer to the stimulation with such pulsatile signal whose amplitude is modulated by the smooth MLDF signals *S*_*i*_(*t*) as *pulsatile MLDF stimulation*.

## 3. Results

### 3.1. Smooth MLDF

**Figure 3:**
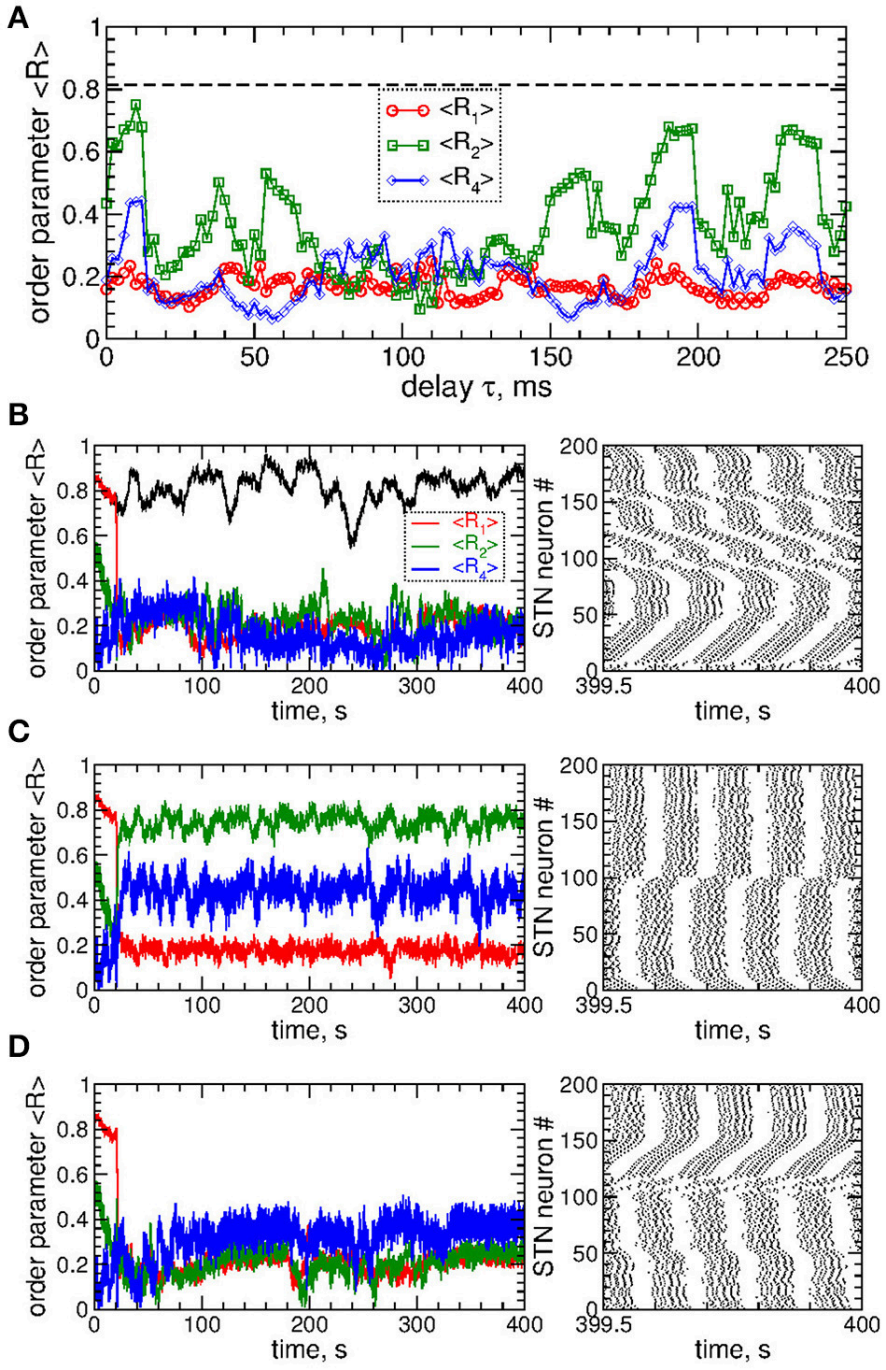
Impact of smooth MLDF on the STN-GPe neurons (Equations 1–4). **(A)** Time-averaged order parameters 〈*R*_1_〉, 〈*R*_2_〉, and 〈*R*_4_〉 (as indicated in the legend) vs. parameter of the feedback delay τ for fixed stimulation intensity *K* = 10. The horizontal dashed line indicates the value of the time-averaged order parameter 〈*R*_1_〉 of the STN neurons without stimulation (*K* = 0). **(B–D)** Corresponding time courses of the order parameters (left plots) and spike raster plots of STN neurons (right plots) for fixed delay **(B)** τ = 72 ms, **(C)** τ = 10 ms, and **(D)** τ = 114 ms. The stimulation starts at *t* = 20 s. In plot **(B)** the time course of the order parameter *R*_1_ of the stimulation-free STN population is also shown (black curve). Coupling *g*_*G*→*S*_ = 1.38 nS/μm^2^.

The impact of the smooth MLDF is illustrated in Figure 3, where synchronized STN neurons are directly stimulated by the smooth feedback signals (Equation 7), i.e., the stimulation currents *I*_stim_ = *S*_*i*_ in Equation (1) for STN neurons. Depending on the parameter of the stimulation delay τ, the stimulation can induce several qualitatively different dynamical regimes as reflected by the values of the order parameters *R*_1_, *R*_2_, and *R*_4_ (Figure 3A). The stimulation can desynchronize the STN neurons, where all order parameters are small as illustrated in Figure 3B for τ = 72 ms. Another stimulation-induced regime is a two-cluster state characterized by small values of *R*_1_ and large values of *R*_2_, see Figure 3A (red circles and green squares). One of such regimes is illustrated in Figure 3C for τ = 10 ms, where the stimulation splits the stimulated neuronal population into two groups of nearly in-phase synchronized neurons which are shifted by approximately 48 ms with respect to each other. These two clusters are thus nearly in anti-phase to each other (LFP oscillation period *T* ≈ 103), which results in a small first order parameter *R*_1_ and large second order parameter *R*_2_ (Figure 3C). Further regimes mimic a four-cluster state, where the fourth order parameter *R*_4_ attains greater values as compared to *R*_1_ and *R*_2_, see Figures 3A,D for τ = 114 ms. Although for such parameters the stimulation clearly splits the stimulated population into four distinct groups phase shifted with respect to each other (with the borders at the lattice coordinates *i* = 1, 50, 100, and 150, see Figure 3D, right panel), the neurons within these clusters can be far from in-phase synchronization. This leads to relatively small values of *R*_4_ and little pronounced four-cluster states.

### 3.2. Pulsatile MLDF

**Figure 4:**
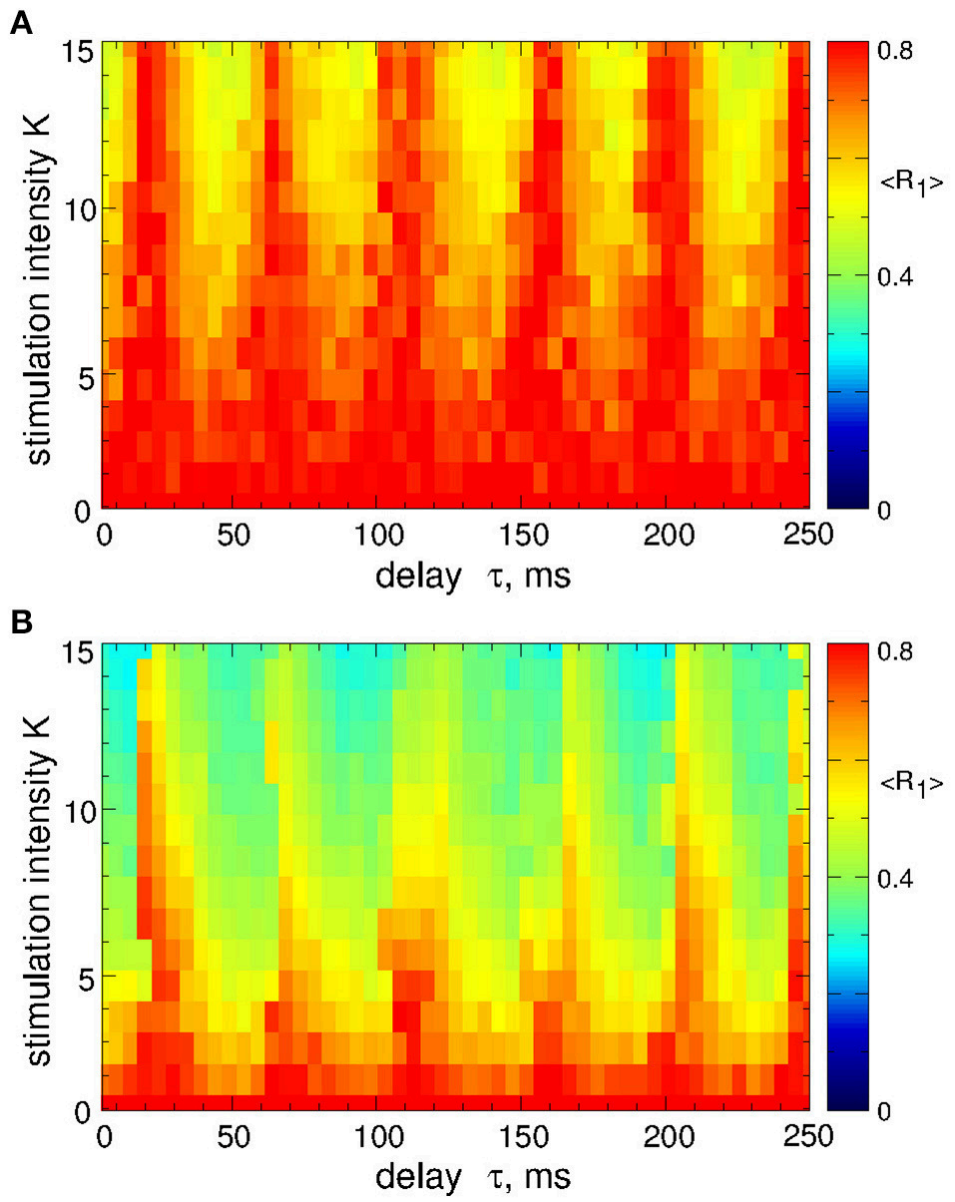
Desynchronization of STN-GPe neurons (Equations 1–4) by pulsatile MLDF. The values of the time-averaged order parameter 〈*R*_1_〉 are encoded in color vs. the stimulation intensity *K* and the stimulation delay τ for the width of the interphase gap **(A)** *GW* = 0 ms and **(B)** 5 ms. Coupling *g*_*G*→*S*_ = 1.38 nS/μm^2^.

**Figure 5:**
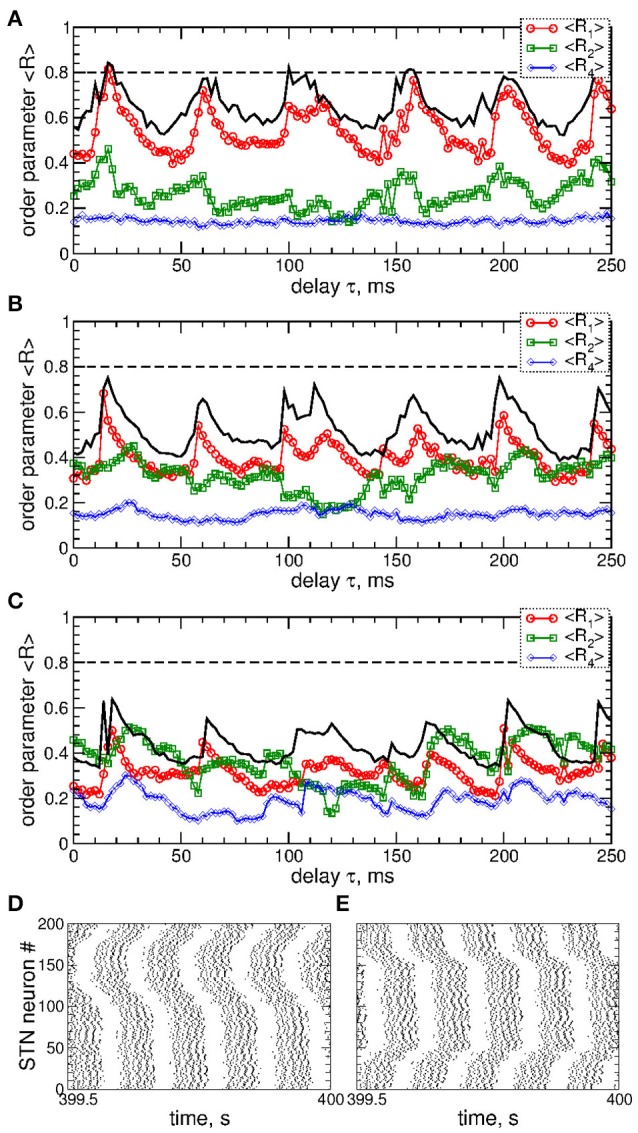
Effect of the interphase gap on the dynamics of STN-GPe neurons (Equations 1–4) induced by pulsatile MLDF. **(A–C)** Time-averaged order parameters 〈*R*_1_〉, 〈*R*_2_〉, and 〈*R*_4_〉 (as indicated in the legends) vs. the stimulation time delay τ for fixed stimulation intensity *K* = 20 and width of the interphase gap **(A)** *GW* = 0 ms, **(B)** 2 ms, and **(C)** 5 ms. The first order parameter 〈*R*_1_〉 for *K* = 10 is also shown for comparison (black solid curves). **(D,E)** Raster plots of the stimulation-induced cluster dynamics for *K* = 20, *GW* = 5 ms, and **(D)** τ = 30 ms and **(E)** τ = 212 ms. Coupling *g*_*G*→*S*_ = 1.38 nS/μm^2^.

As mentioned above, the safety requirements for the electrical stimulation of neuronal tissue may be violated for direct electrical stimulation with smooth and slowly oscillating feedback signals. We thus utilize a pulsatile stimulation protocol for MLDF, see section Methods and Figure 2B. Such a pulsatile MLDF stimulation is administered to synchronized STN neurons (Figure 1) for different values of the stimulation parameters τ and *K*. The time-averaged first order parameter of the obtained stimulation-induced regimes is depicted in Figure 4 in color vs. parameters (τ, *K*) for width of the interphase gap *GW* = 0 ms (Figure 4A) and 5 ms (Figure 4B). As compared to the case of smooth MLDF stimulation (Figure 3) the first order parameter *R*_1_ exhibits much more pronounced alterations when the delay parameter τ is varied such that several desynchronization regions emerge in the parameter space characterized by small values of *R*_1_.

When stimulation pulses include an interphase gap (Figure 2B) the desynchronizing effect of the pulsatile MLDF stimulation can significantly be improved as illustrated in Figure 4B for the interphase gap *GW* = 5 ms. In fact, for vanishing interphase gap the desynchronizing effect is only moderate (Figure 4A). The structure of the parameter space with desynchronization regions is preserved, but the extent of the stimulation-induced desynchronization is enhanced, as reflected by the values of the first order parameter, i.e., the values of *R*_1_ get smaller, compare Figure 4A and Figure 4B. This indicates a favorable effect of the interphase gap on the desynchronization outcome of the pulsatile MLDF.

A detailed consideration by calculating all order parameters *R*_1_, *R*_2_, and *R*_4_ reveals that pulsatile MLDF stimulation with zero gap does not induce any kind of clustering. This is implied by relatively large values of the first order parameter *R*_1_ and small values of the other order parameters, see Figure 5A. As mentioned above (Figure 4), an increase of the interphase gap results in a decrease of the first order parameter, which can also be observed in Figures 5A–C (red circles). Simultaneously the other order parameters increase. For example, for the stimulation intensity *K* = 20, delay τ = 30 ms, and interphase gaps *GW* = 0 ms, 2 ms, and 5 ms the order parameters *R*_1_ ≈ 0.54, 0.4, 0.3, *R*_2_ ≈ 0.23, 0.41, 0.5, and *R*_4_ ≈ 0.14, 0.16, 0.26, respectively. A relatively large *R*_2_ and small *R*_1_ are indicative for a two-cluster state. The pattern of the neuronal firing of STN neurons for such parameters is illustrated in Figure 5D, where two groups of neurons (clusters) with different patterns of activity can be distinguished for neuron indices *i* < 100 and *i* > 100. Another example of a two-cluster state is illustrated in Figure 5E for the gap width *GW* = 5 ms and delay τ = 212 ms from another parameter region of a two-cluster regime of large *R*_2_ and small *R*_1_, see Figure 5C.

We compare the desynchronizing impact of the pulsatile MLDF to that of pulsatile linear delayed feedback (LDF). The smooth and pulsatile LDF administered to synchronized STN neurons has been investigated in Popovych et al. (2017a,b) together with smooth and pulsatile nonlinear delayed feedback (NDF). The feedback signal *S*(*t*) of the differential LDF can be obtained as (Rosenblum and Pikovsky, 2004a,b):

(8) $$\begin{array}{l} S\left(t\right)=K\left(x\left(t-\tau \right)-x\left(t\right)\right), \end{array}$$

where, as before, the variable *x*(*t*) is a filtered LFP and calculated by means of Equation (6), i.e., $x\left(t\right)=\dot{u}$, and *K* and τ are the parameters of the stimulation intensity and delay, respectively. The smooth feedback signal *S*(*t*) of LDF is then used to modulate the amplitude of the stimulation pulses as discussed above, where we assume that all STN neurons receive the same stimulation current *I*_stim_ depicted in Figure 2B.

**Figure 6:**
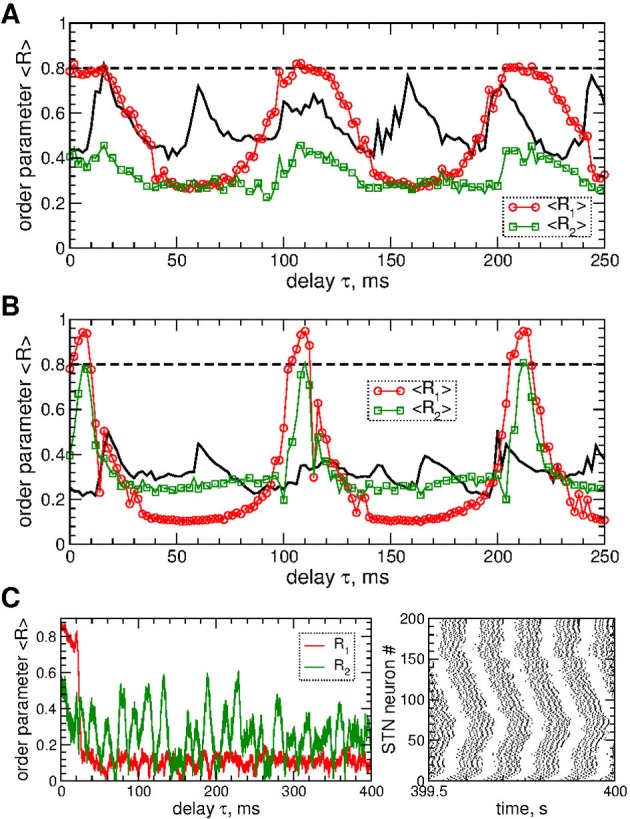
Desynchronization of STN-GPe neurons (Equations 1–4) by pulsatile LDF. **(A,B)** Time-averaged order parameters 〈*R*_1_〉 and 〈*R*_2_〉 (as indicated in the legend) of the STN neurons stimulated by pulsatile LDF vs. the stimulation delay τ for fixed stimulation intensity *K* = 20 and interphase gap **(A)** *GW* = 0 ms and **(B)** 5 ms. The first order parameter 〈*R*_1_〉 for pulsatile MLDF stimulation is also shown for comparison (black solid curves, see Figures 5A,C, red circles). **(C)** Example of the time courses of the order parameters *R*_1_ and *R*_2_ (left plot) and the corresponding spike raster plot of STN neurons (right plot) for pulsatile LDF stimulation for fixed *K* = 20, τ = 60 ms, and *GW* = 5 ms (from plot **B**). Coupling *g*_*G*→*S*_ = 1.38 nS/μm^2^.

STN neurons can be desynchronized by the pulsatile LDF stimulation as illustrated in Figure 6. The parameter space of the pulsatile LDF contains large desynchronization regions, where the first order parameter *R*_1_ exhibits pronounced minima (Figures 6A,B, red circles). An interphase gap of a finite width can enhance the desynchronizing effect of pulsatile LDF, where the desynchronization regions are enlarged and deepened such that the stimulation induces stronger desynchronization as reflected by smaller values of *R*_1_ for larger interphase gap, compare Figure 6A and Figure 6B (red circles). The other order parameters of higher degree are however not significantly affected by the values of the interphase gap. For example, the values of the second order parameter *R*_2_ are nearly preserved within the desynchronization regions when the width of the interphase gap increases from *GW* = 0 to 5 ms, compare Figure 6A and Figure 6B (green squares). Albeit the suppressed second order parameter *R*_2_ can still be somewhat larger than the first order parameter *R*_1_ (Figure 6B), the stimulated neurons do not exhibit any consistent clustering as illustrated in Figure 6C. This indicates that the pulsatile LDF stimulation does not induce clustering among stimulated STN neurons for any widths of the interphase gap, which is different to the impact of the pulsatile MLDF, see Figure 5.

**Figure 7:**
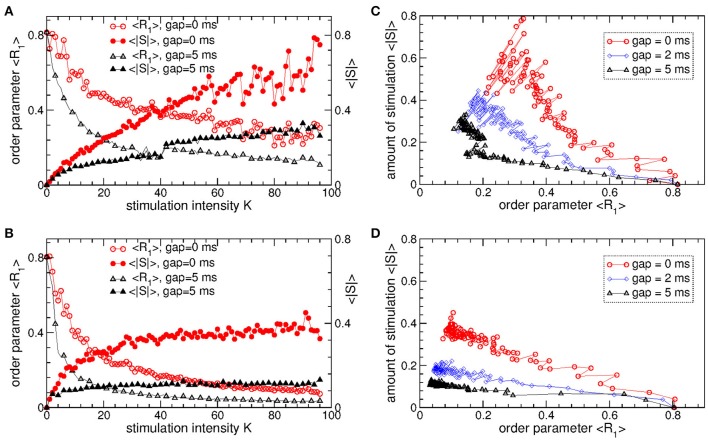
Desynchronizing outcome of pulsatile MLDF and LDF stimulations administered to STN-GPe neurons (Equations 1–4). **(A,B)** Time-averaged first order parameter 〈*R*_1_〉 and the absolute value 〈|*S*|〉 of the feedback signal **(A)** Equation (7) of MLDF and **(B)** Equation (8) of LDF vs. parameter *K* of the stimulation intensity for two widths *GW* = 0 ms and 5 ms of the interphase gap as indicated in the legends. The scale of 〈|*S*|〉 is indicated on the right vertical axis. **(C,D)** Administered amount of the stimulation as given by the values of 〈|*S*|〉 vs. the reached extent of the stimulation-induced desynchronization as given by values of 〈*R*_1_〉 for **(C)** pulsatile MLDF and **(D)** pulsatile LDF for different width of the interphase gap as indicated in the legends. Stimulation delay **(A,C)** τ = 90 ms for MLDF and **(B,D)** τ = 60 ms for LDF. Coupling *g*_*G*→*S*_ = 1.38 nS/μm^2^.

The pulsatile LDF is more efficient in inducing desynchronization than the pulsatile MLDF. For the same stimulation intensity *K* and width of the interphase gap, the pulsatile LDF can induce much stronger desynchronization than the pulsatile MLDF as given by the values of the first order parameter *R*_1_ within desynchronization regions, see Figures 6A,B and compare black solid curves (*R*_1_ for MLDF) to red circles (*R*_1_ for LDF). For a more detailed comparison, we fix optimal stimulation delay τ = 90 ms for pulsatile MLDF and τ = 60 ms for pulsatile LDF, where the stimulation induces strongest desynchronization, see Figures 4–6, and increase the stimulation intensity *K*. We find that both pulsatile MLDF and LDF stimulations with larger intensity can induce stronger desynchronization, and the first order parameter *R*_1_ decreases when *K* increases, see Figure 7A for MLDF and Figure 7B for LDF (empty symbols). We also observe that *R*_1_ decreases much faster with increasing *K* and can reach much smaller values for pulsatile LDF than for MLDF for the same range on the stimulation intensity.

An important characteristics of the stimulation of the neuronal tissue is the amount of the administered stimulation. We thus estimate it for the considered feedback stimulations by the time-averaged absolute value 〈|*S*|〉 of the smooth feedback signals (Equation 7) for MLDF and (Equation 8) and LDF. The amount of the administered stimulation is depicted in Figure 7A for MLDF and Figure 7B for LDF (filled symbols) vs. parameter *K* of the stimulation intensity. When *K* increases, the amount of the administered stimulation also increases such that stronger desynchronization can be obtained at stronger stimulation. Since the amplitude of the feedback signals depends on the amplitude of the LFP, it also inversely relates to the extent of the stimulation-induced desynchronization. For a given value of the stimulation intensity *K*, the amount of the administered stimulation 〈|*S*|〉 will be smaller if the stimulation-induced desynchronization is stronger, i.e., when the order parameter *R*_1_ and amplitude of the LFP are smaller. Therefore, for the considered range of parameter *K*, 〈|*S*|〉 increases more slowly for pulsatile LDF than for pulsatile MLDF with increasing stimulation intensity, see Figures 7A,B (filled symbols). For both stimulation methods larger interphase gap leads to better desynchronization and smaller amount of the administered stimulation.

The latter claim is also supported by Figures 7C,D, where the amount of the administered stimulation 〈|*S*|〉 is depicted vs. the extent of the stimulation-induced desynchronization as reflected by the first order parameter *R*_1_. As follows, the same extent of desynchronization can be obtained at smaller amount of the administered stimulation for larger interphase gap. In such a way we can compare the efficacy in inducing desynchronization of MLDF and LDF methods by comparing the amount of the administered stimulation necessary to achieve the same extent of the stimulation-induced desynchronization. Comparing the depicted data for pulsatile MLDF (Figure 7C) and pulsatile LDF (Figure 7D) we conclude that the pulsatile LDF is more effective in inducing desynchronization than pulsatile MLDF, where desynchronization can be obtained for much smaller amount of the administered stimulation.

**Figure 8:**
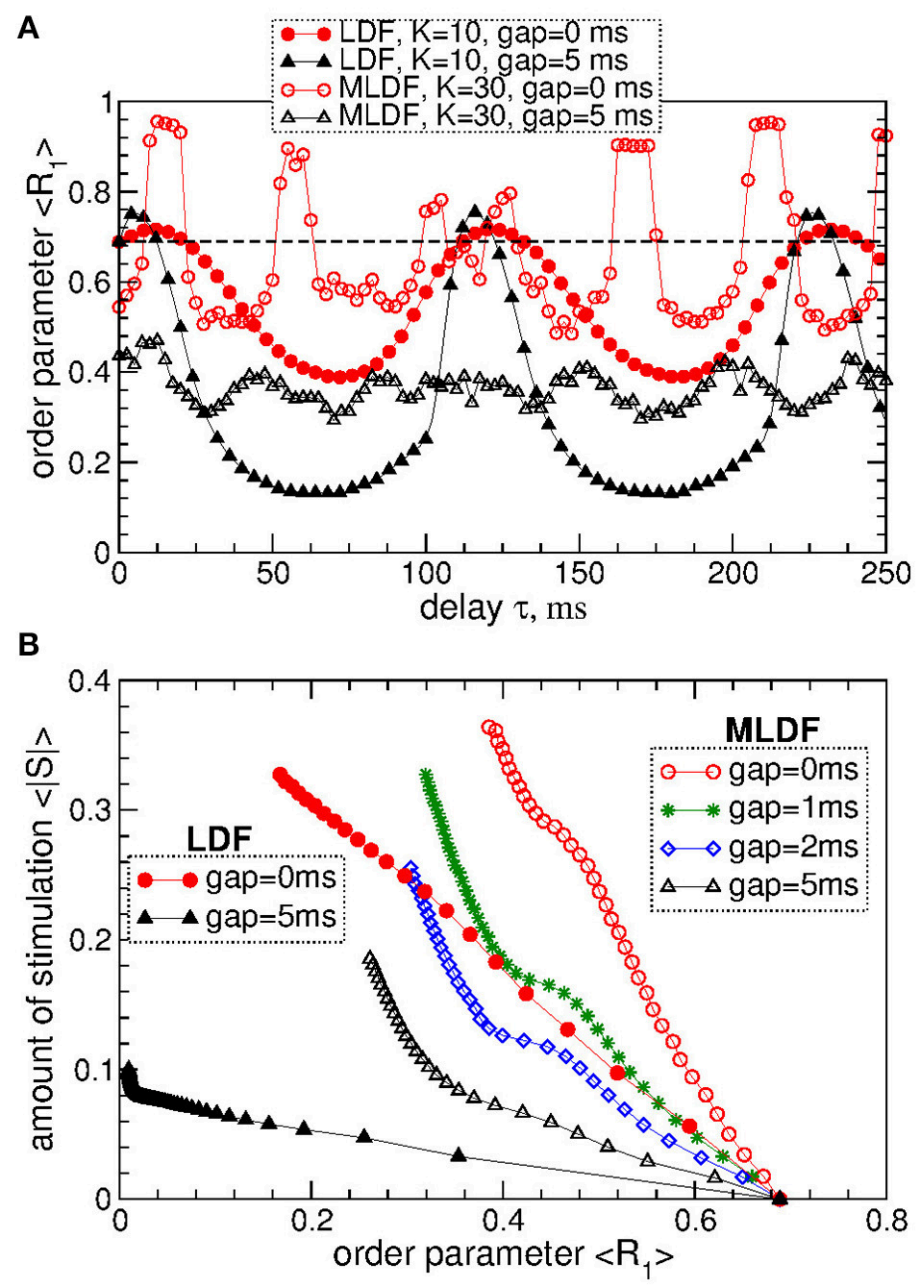
Desynchronization of periodically synchronized STN-GPe neurons (Equations 1–4) by pulsatile MLDF and LDF stimulations. **(A)** Time-averaged order parameter 〈*R*_1_〉 of the STN neurons stimulated by pulsatile MLDF with *K* = 30 and pulsatile LDF with *K* = 10 vs. the stimulation delay τ for two widths of the interphase gap *GW* = 0 ms and 5 ms as indicated in the legend. **(B)** Administered amount of the stimulation as given by the values of 〈|*S*|〉 vs. the reached extent of the stimulation-induced desynchronization as given by values of 〈*R*_1_〉 for pulsatile MLDF (empty symbols) with τ = 50 ms and LDF (filled symbols) with τ = 70 ms. The width of the interphase gap is indicated in the legend. Coupling *g*_*G*→*S*_ = 1.7 nS/μm^2^.

The above results obtained for the synchronized regime of STN-GPe neurons with irregular interburst intervals (Figure 1 for coupling *g*_*G*→*S*_ = 1.7 nS/μm^2^) are preserved for other parameters and synchronized regimes in the considered populations of the STN-GPe neurons. We consider, for instance, stronger coupling *g*_*G*→*S*_ = 1.7 nS/μm^2^, where the STN-GPe neurons are periodically synchronized, see Figure 1A (red curve) and Figure 1D. The desynchronizing effect of the pulsatile MLDF and LDF on the periodically synchronized STN-GPe neurons is illustrated in Figure 8A. The structure of the parameter space is preserved for both stimulation methods except for that the pulsatile MLDF induces a somewhat weaker desynchronization as compared to the above case of a weaker coupling. The clustering induced by the pulsatile MLDF for the considered stronger coupling also becomes less pronounced. The efficacy of the pulsatile MLDF and LDF in inducing desynchronization is compared in Figure 8B, where the amount of the administered stimulation 〈|*S*|〉 is depicted vs. the time-averaged first order parameter 〈*R*_1_〉. As for the case of the irregular synchronization for weaker coupling (Figure 7), the interphase gap has the same favorable impact on the stimulation outcome, and the pulsatile LDF is apparently superior to MLDF and can induce stronger desynchronization for smaller amount of administered stimulation.

### 3.3. Differential MLDF

**Figure 9:**
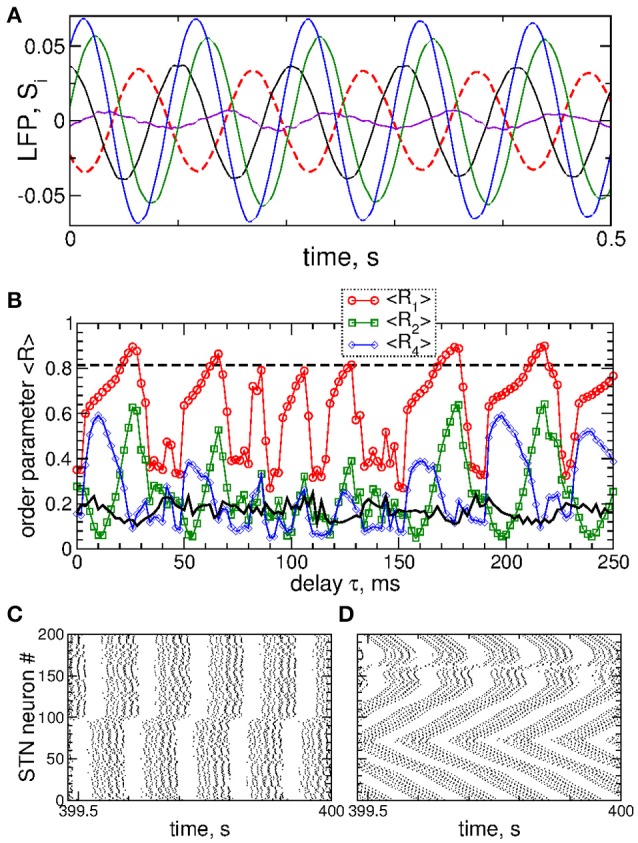
Impact of the differential smooth MLDF on collective dynamics of STN-GPe neurons (Equations 1–4). **(A)** Example of the feedback signals *S*_*i*_ of the differential MLDF (Equation 9) for delay τ = 90 ms. The filtered LFP is depicted by dashed red curve. **(B)** Time-averaged order parameters 〈*R*_1_〉, 〈*R*_2_〉, and 〈*R*_4_〉 (as indicated in the legend) vs. parameter of the feedback delay τ for fixed stimulation intensity *K* = 10. The first order parameter 〈*R*_1_〉 of the STN neurons for direct smooth MLDF stimulation (Equation 7) is also shown by the black solid curve for comparison (copied from Figure 3A, red circles). The horizontal dashed line indicates the value of 〈*R*_1_〉 without stimulation (*K* = 0). **(C,D)** Spike raster plots of STN neurons for fixed stimulation intensity *K* = 10 and delays **(C)** τ = 10 ms and **(D)** τ = 90 ms. Coupling *g*_*G*→*S*_ = 1.38 nS/μm^2^.

To overcome the limitations of pulsatile MLDF revealed above, we suggest to use a differential MLDF. The feedback signals are constructed by analogy with the differential LDF (Equation 8) and read.

(9) $$\begin{array}{l} S_{i}\left(t\right)=K\cdot \left(x\left(t-\tau_{i}\right)-x\left(t\right)\right),i=1,2,3,4, \end{array}$$

where the signal *x*(*t*) is the filtered LFP from Equation (6), and the delays are as in Equation (7). As before, we reverse the polarity of the two feedback signals, such that *S*_3_ = *K* · (−*x*(*t* − τ_1_) − *x*(*t*)) and *S*_4_ = *K* · (−*x*(*t* − τ_2_) − *x*(*t*)). An example of the feedback signals *S*_*i*_ is illustrated in Figure 9A. As for the case of direct MLDF (Equation 7), the feedback signals of the differential MLDF are time shifted with respect to each other, but, in contrast to the direct MLDF, they may however have very different amplitude.

Stimulation by differential smooth MLDF can perturb the neuronal synchronization of the stimulated STN neurons as illustrated in Figures 9B–D, where the time-averaged order parameters 〈*R*_1_〉, 〈*R*_2_〉, and 〈*R*_4_〉 are plotted vs. the stimulation delay τ. Based on the values of the first order parameter *R*_1_, smooth differential MLDF is less effective in inducing desynchronization than smooth direct MLDF, compare values of *R*_1_ in Figure 9B of differential MLDF (red circles) to those of direct MLDF (black curve). For differential MLDF large values of the other order parameters are accompanied by large values of *R*_1_. This indicates that clusters of a possible clustered state are not equidistantly spaced over the oscillation period. Indeed, this is illustrated in Figure 9C for delay τ = 10 ms, where the fourth order parameter *R*_4_ attains maximal values, see Figure 9B. In this example the stimulation divides the neurons into two groups, where the burst onsets of the two clusters are time shifted with respect to each other by Δ*T* ≈ 26 ms (Figure 9C). Such firing patterns result in large values of the order parameters *R*_1_ and *R*_4_, but in small values of *R*_2_ as depicted in Figure 9B for the mentioned value of the stimulation delay. The best desynchronization can be achieved, for example, at τ = 90 ms as reflected by small values of *R*_1_ (Figure 9B). The corresponding firing pattern of STN neurons is illustrated in Figure 9D.

**Figure 10:**
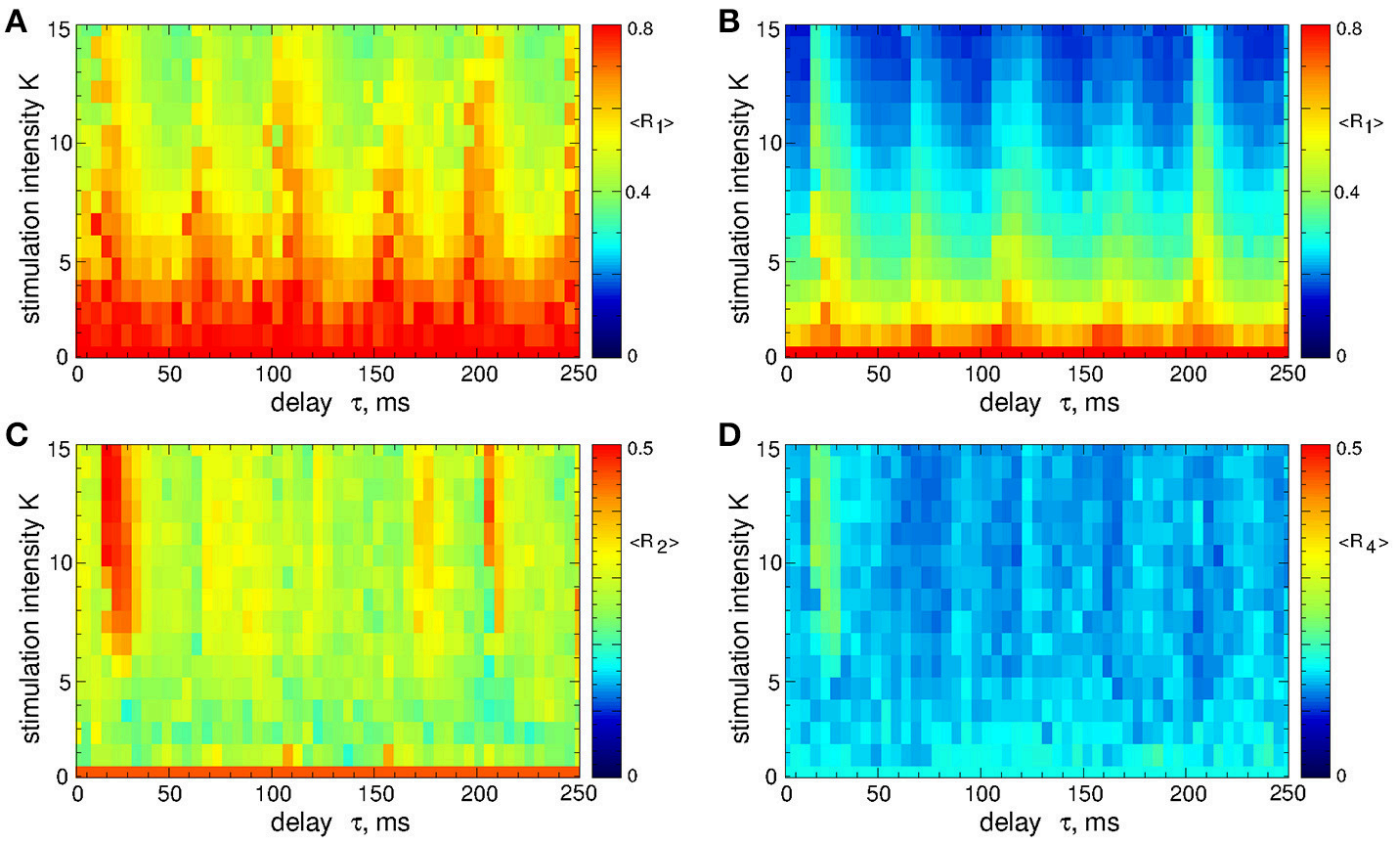
Desynchronization of STN-GPe neurons (Equations 1–4) by the differential pulsatile MLDF (Equation 9). The values of the time-averaged order parameters **(A,B)** 〈*R*_1_〉, **(C)** 〈*R*_2_〉, and **(D)** 〈*R*_4_〉 are depicted in color vs. the stimulation intensity *K* and delay τ for the width of the interphase gap **(A)** *GW* = 0 ms and **(B–D)** 5 ms. Coupling *g*_*G*→*S*_ = 1.38 nS/μm^2^.

In contrast to the stimulation with smooth signals, the differential pulsatile MLDF can induce stronger desynchronization as compared to the direct pulsatile MLDF. Two-parameter diagrams for differential pulsatile MLDF, where the order parameters are depicted in color in the (τ, *K*)-parameter plane, are shown in Figure 10. For the same range of the stimulation parameters the first order parameter *R*_1_ (Figures 10A,B) exhibits smaller values as compared to those depicted in Figure 4 for direct pulsatile MLDF. Interphase gap enhances the extent of the stimulation-induced desynchronization, compare Figure 10A and Figure 10B. For large interphase gap the (τ, *K*)-parameter plane contains a narrow region around τ ≈ 15 ms, where the second order parameter *R*_2_ is relatively large (Figure 10C). For such parameters of the best two-cluster regime, the pattern of the neuronal firing looks similar to that in Figure 5D, i.e., there are no pronounced two- and four-cluster states.

**Figure 11:**
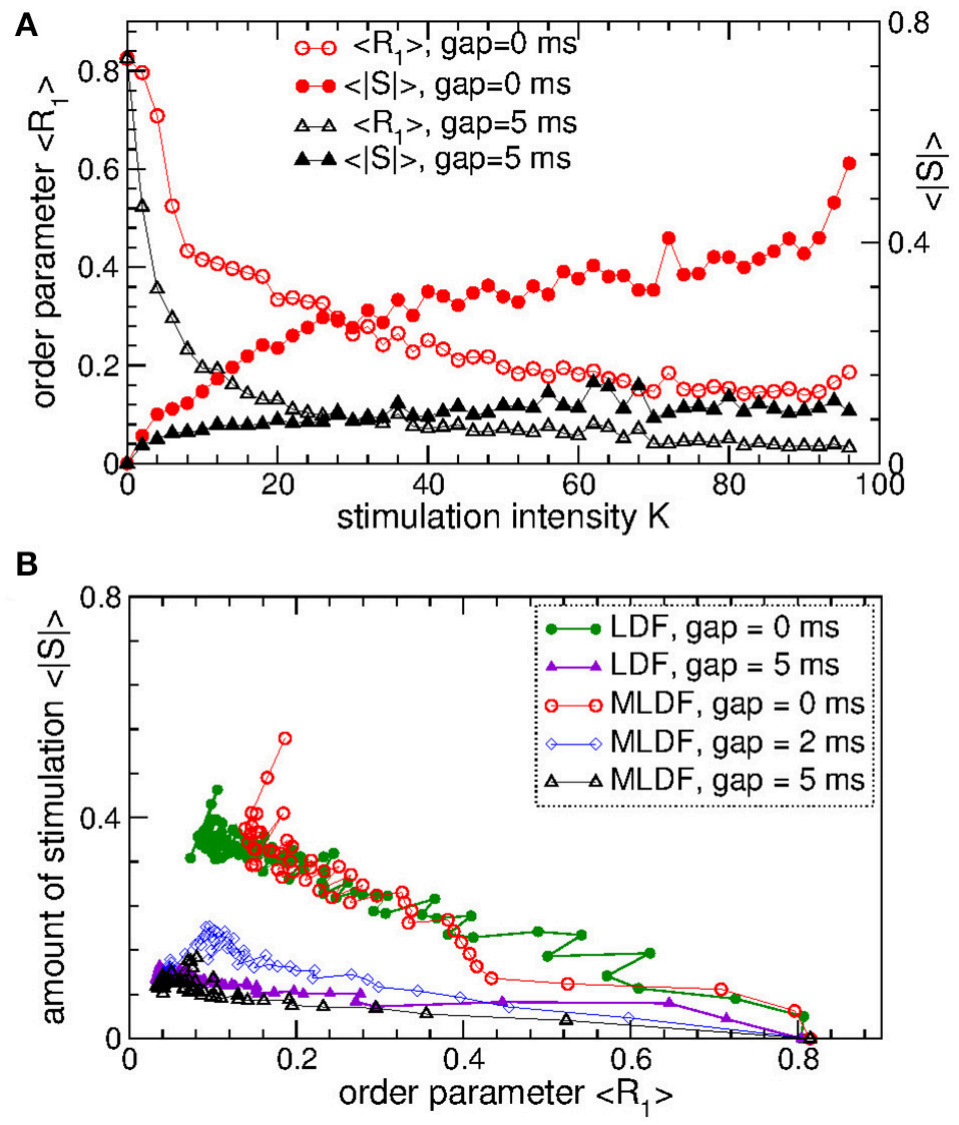
Stimulation outcome of the differential pulsatile MLDF stimulation administered to STN-GPe neurons (Equations 1–4). **(A)** Time-averaged first order parameter 〈*R*_1_〉 and the averaged absolute value 〈|*S*|〉 of the feedback signals (Equation 9) of differential MLDF vs. parameter *K* of the stimulation intensity for two widths *GW* = 0 ms and 5 ms of the interphase gap as indicated in the legend. The scale of 〈|*S*|〉 is indicated on the right vertical axis. **(B)** Administered amount of the stimulation as given by the values of 〈|*S*|〉 vs. the reached extent of the stimulation-induced desynchronization as given by values of 〈*R*_1_〉 for differential pulsatile MLDF (empty symbols) for delay τ = 90 ms and pulsatile LDF (filled symbols, copied from Figure 7D for comparison) for delay τ = 60 ms for different width of the interphase gap as indicated in the legend. Coupling *g*_*G*→*S*_ = 1.38 nS/μm^2^.

To evaluate the stimulation outcome of the differential pulsatile MLDF we calculate the amount of the administered stimulation 〈|*S*|〉 that is approximated by the average of the absolute values of the MLDF feedback signals (Equation 9). In Figure 11A 〈|*S*|〉 is depicted together with the extent of the stimulation-induced desynchronization as given by the values of the first order parameter 〈*R*_1_〉 vs. parameter of the stimulation intensity *K*. For the differential pulsatile MLDF, the order parameter 〈*R*_1_〉 decreases much faster than for the direct pulsatile MLDF, and, as a results, 〈|*S*|〉 increases more slowly, compare Figure 11A to Figure 7A. The efficacy of the differential pulsatile MLDF in inducing desynchronization is comparable with that of the pulsatile LDF, see Figure 7B. Indeed, this conclusion apparently follows from Figure 11B, where a given extent of the stimulation-induced desynchronization as given by values of *R*_1_ can be obtained at approximately the same amount of the administered stimulation for differential pulsatile MLDF (Figure 11B, empty symbols) and for pulsatile LDF (Figure 11B, filled symbols).

**Figure 12:**
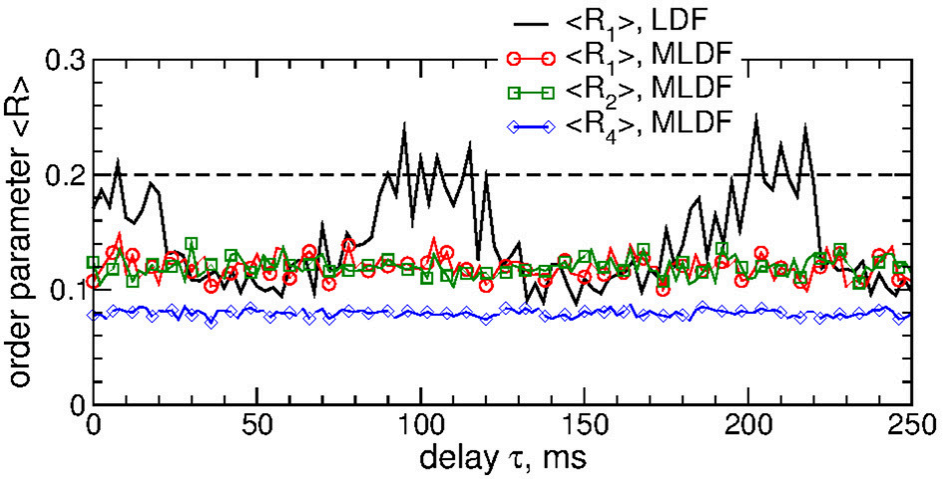
Impact of the differential pulsatile MLDF on collective dynamics of weakly coupled and weakly synchronized STN-GPe neurons (Equations 1–4). Time-averaged order parameters 〈*R*_*i*_〉, *i* = 1, 2, 4, are plotted (as indicated in the legend) vs. parameter of the feedback delay τ for fixed stimulation intensity *K* = 10. The first order parameter 〈*R*_1_〉 of the STN neurons stimulated by pulsatile LDF is also shown by the black solid curve for comparison. The horizontal dashed line indicates the value of 〈*R*_1_〉 without stimulation (*K* = 0), see Figure 1A (black curve). Coupling *g*_*G*→*S*_ = 1.28 nS/μm^2^ and interphase gap *GW* = 5 ms.

We also verified that differential pulsatile MLDF stimulation is robust with respect to the extent of the initial synchronization in the stimulated neuronal population. If the stimulation is administered to weakly synchronized neurons as, for example, for the coupling parameter *g*_*G*→*S*_ = 1.28 nS/μm^2^, see Figure 1, the synchronization can further be suppressed by differential pulsatile MLDF practically irrespective of the values of the delay parameter τ, see Figure 12. Such a reduction of an already weak and intermittent neuronal synchronization is comparable with or even slightly better than that of pulsatile LDF, see also Popovych et al. (2017a,b).

## 4. Discussion

Multisite linear delayed feedback (MLDF) has been suggested for control of neuronal synchronization patterns (Hauptmann et al., 2005a,b, 2007a,b; Popovych et al., 2006; Omel'chenko et al., 2008). As shown computationally, stimulation by MLDF can suppress synchronization in a stimulated neuronal population and was, hence, suggested for counteracting abnormal neuronal synchronization characteristic for several neurological disorders (Hauptmann et al., 2005a,b, 2007b; Popovych et al., 2006). The desynchronization induced by MLDF stimulation was found to be accompanied by the emergence of several interacting clusters of neurons equidistantly distributed over the oscillation period and space. Depending on the stimulation setup and parameters the stimulation-induced spatio-temporal patters can consist of two or four clusters, for example, for four-site MLDF stimulation (Hauptmann et al., 2005a,b, 2007a,b; Popovych et al., 2006; Omel'chenko et al., 2008). These properties of MLDF made the method appropriate for the control of spatio-temporal patterns of neuronal activity, for example, for regulating activity of central pattern generator (CPG) in case of its malfunction as suggested in Hauptmann et al. (2007a,b) and Omel'chenko et al. (2008).

In this study we adapted the MLDF technique for electrical stimulation of the neuronal tissue. As mentioned above, direct electrical stimulation with smooth feedback signals may violate safety aspects like charge density limits (Harnack et al., 2004; Kuncel and Grill, 2004; Merrill et al., 2005). The feedback signals are slow, so that an irreversible charge can be deposited into the neuronal tissue during the comparably long feedback stimulation periods, which can exceed safety limits. We resolved this problem and showed that the demand-controlled character and desynchronizing impact of the MLDF feedback technique can be preserved together with gaining the advantages of the pulsatile HF DBS signal with the charge-balanced property. For this, the slow feedback signal is used to modulate the amplitude of the HF train of charge-balanced pulses of HF DBS. We computationally illustrated the desynchronizing properties of smooth and pulsatile MLDF in a network of STN-GPe neurons suggested to model parkinsonian neuronal dynamics (Terman et al., 2002; Rubin and Terman, 2004).

We showed that both smooth and pulsatile MLDF stimulation can suppress neuronal synchronization in the stimulated STN neurons. While smooth MLDF could induce relatively well pronounced two-cluster states in some parameter ranges, the expected four-cluster states were only weakly expressed and could be observed in a limited parameter range. For pulsatile MLDF we found that interphase gap in the stimulation pulses could significantly enhance the desynchronizing impact of the stimulation. The clustering state was observed for large interphase gap only, where some two-cluster states could be induced by the stimulation for some selected parameter values. We thus conclude that the pulsatile MLDF is mostly a desynchronizing stimulation rather than inducing coordinated spatio-temporal clustering patterns. We however showed that the efficacy of the pulsatile MLDF in inducing desynchronization was much lower than that of the pulsatile LDF. Therefore, in the standard realization the pulsatile MLDF can be suggested as effective method neither for desynchronization nor for the control of spatio-temporal clustering patterns.

We therefore proposed to use a differential pulsatile MLDF. Such a modified pulsatile MLDF turned out to hardly induce clustering for any width of the interphase gap. Nevertheless, we showed that differential pulsatile MLDF can effectively and robustly desynchronize the stimulated neurons. We verified that the efficacy of the differential pulsatile MLDF in inducing desynchronization is comparable with that of the pulsatile LDF, and we suggest this technique for closed-loop desynchronizing DBS together with pulsatile LDF and NDF investigated in recent papers (Popovych et al., 2017a,b). The differential pulsatile MLDF is robust with respect to variations of the stimulation parameters, in particular, if initially weakly synchronized neuronal populations need to be further desynchronized. However, MLDF requires several stimulation sites to be placed in the neuronal target population. In the case of small targets this might be difficult, so that single-site stimulation techniques such as pulsatile LDF and NDF may be more appropriate in those cases. It would be interesting to investigate how the effectiveness and efficacy of pulsatile NDF stimulation are affected if a multisite stimulation protocol is adapted for this method. It would also be interesting to use a realistic 3-Dim reconstruction of the target neuronal structures, e.g., STN and GPe, as well as localization of the stimulation sites within these structures to explore the spatio-temporal patterns induced by a multisite stimulation (Ebert et al., 2014), which however essentially requires the usage of a supercomputer.

However, as a word of caution, it should be noted that the approach presented in this study relies on the assumption that abnormal neuronal synchrony is recordable and represents the patient's individual symptoms in a sufficient manner, like a biomarker (Beudel and Brown, 2016; Kühn and Volkmann, 2017). For instance, it is doubtful that beta band oscillations might be a biomarker-like feedback signal (Özkurt et al., 2011; Johnson et al., 2016; Kühn and Volkmann, 2017). Beta band oscillations are no stand-alone oscillations, but interact with brain oscillations in other frequency bands under both physiological (Yanagisawa et al., 2012) and pathological (Yang et al., 2014; Beudel and Brown, 2016) conditions. Different PD phenotypes might require different biomarkers, since the amplitude of beta band oscillations may decrease during tremor epochs in tremor dominant PD patients (Quinn et al., 2015). Changes of the amplitude of abnormal brain oscillations in the course of physiological processes have to be taken into account, too. For instance, in an MPTP monkey study HF DBS and closed-loop DBS (CL-DBS) reduced rigidity to a comparable extent, where CL-DBS reduced the DBS ON time by approx. 50% (Johnson et al., 2016). However, only HF DBS improved bradykinesia during a cued reaching task, likely because the amplitude of beta band oscillations was reduced related to the reaching process, in this way reducing the extent of the presumed biomarker (Johnson et al., 2016). Also, beta band oscillations need not be entirely pathological. Rather activity in the beta frequency range might be key for compensatory purposes, as demonstrated in an MPTP monkey study with sensorimotor rhythm neurofeedback (Philippens et al., 2017).

Apart from merely reducing the stimulation current, differential pulsatile MLDF may be beneficial because of its specifically desynchronizing effect. As shown computationally in the context of Coordinated Reset (CR) stimulation (Tass, 2003), desynchronizing stimulation may cause an anti-kindling, where abnormal synaptic connectivity and neuronal synchrony can be unlearned, ultimately leading to sustained desynchronizing effects (Tass and Majtanik, 2006; Popovych and Tass, 2012). In accordance with these theoretical predictions, long-lasting therapeutic effects were demonstrated in pre-clinical studies in MPTP monkeys with CR-DBS (Tass et al., 2012b; Wang et al., 2016) as well as in a clinical proof of concept study with CR-DBS in Parkinson's patients (Adamchic et al., 2014). Analogously, long-lasting therapeutic effects were observed in a proof of concept study with acoustic CR stimulation in tinnitus patients (Tass et al., 2012a) as well as in a first in man study with vibrotactile CR stimulation in patients with Parkinson's disease (Syrkin-Nikolau et al., 2018).

The requirements for CR-DBS and MLDF are quite different. CR stimulation can be administered in open loop as well as closed loop, e.g., demand-controlled manner (Tass, 2003). In particular, CR stimulation does not require a feedback signal. So far, pre-clinical (Tass et al., 2012b; Wang et al., 2016) and clinical (Adamchic et al., 2014) proof of concept of CR-DBS were obtained with open loop CR-DBS. In contrast, MLDF requires a reliably measurable clean biomarker signal sufficiently representing the amount of abnormal synchronization. Despite first positive results (Little et al., 2013; Rosa et al., 2015), several findings indicate that beta-band STN LFP does not provide a reliable biomarker (Özkurt et al., 2011; Quinn et al., 2015; Johnson et al., 2016; Kühn and Volkmann, 2017; Philippens et al., 2017), see above. In addition, MLDF requires a challenging registration-stimulation setup: An LFP signal, representative for the entire neuronal target population, has to be measured at one site, while stimuli have to be delivered to different sites of the target population. Because of stimulation artifacts this might be difficult. However, to overcome this limitation, alternatively, one might try to measure a representative LFP and stimulate different parts of fibers projecting on the target population. One could also separate stimulation and recording in time (Ratas and Pyragas, 2014).

## Author contributions

PT conceived HFS amplitude modulation by feedback. OP performed the experiments, analyzed the data, and prepared the initial draft of the manuscript. PT contributed to the numerical analysis and extended the manuscript. All authors reviewed the manuscript.

### Conflict of interest statement

The authors declare that the research was conducted in the absence of any commercial or financial relationships that could be construed as a potential conflict of interest.
