# Supplementary material for: Multisite Delayed Feedback for Electrical Brain Stimulation
Source: Front Physiol. 2018 Feb 1;9:46. doi: 10.3389/fphys.2018.00046 (PMC5799832; doi:10.3389/fphys.2018.00046)
Supplement: Supplementary file 1 [file Table1.PDF]

Supplementary Material:

Multisite delayed feedback for electrical brain stimulation

Oleksandr V. Popovych* and Peter A. Tass
*Correspondence:
Oleksandr V. Popovych
o.popovych@fz-juelich.de

The parameter values of the neuronal model (1) - (4) are listed in Supplementary Table S1. The model was originally introduced in paper (Terman et al., 2002), and the values of parameters mostly coincide with those from the papers (Terman et al., 2002; Rubin and Terman, 2004; Park et al., 2011) and are taken to demonstrate the synchronous bursting dynamics of the STN neurons, which is a hallmark of the pathological parkinsonian state (Hammond et al., 2007; Benabid et al., 2009).

Table S1. Parameter set of the considered model of STN-GPe network (1) - (4).

Parameter	STN	GPe	Units	Parameter	STN	GPe	Units
$g_{\rm L}$	2.25	0.1	nS/μm^2	θ_m	-30.0	-37.0	mV
$g_{\rm K}$	40	30	nS/μm^2	θ_h	-39.0	-58.0	mV
$g_{\rm Na}$	50	120	nS/μm^2	θ_n	-32.0	-50.0	mV
$g_{\rm T}$	0.5	0.5	nS/μm^2	θ_r	-67.0	-70.0	mV
$g_{\rm Ca}$	0.5	0.15	nS/μm^2	θ_a	-63.0	-57.0	mV
$g_{\rm AHP}$	9.0	30	nS/μm^2	θ_b	0.4	–	
$v_{\rm L}$	-60.0	-55.0	mV	θ_s	-39.0	-35.0	mV
$v_{\rm K}$	-80.0	-80.0	mV	θ_h^τ	-57.0	-40.0	mV
$v_{\rm Na}$	55.0	55.0	mV	θ_n^τ	-80.0	-40.0	mV
$v_{\rm Ca}$	140.0	120.0	mV	θ_r^τ	68.0	–	mV
τ_h^1	500.0	0.27	ms	θ_g^H	-39.0	-57.0	mV
τ_n^1	100.0	0.27	ms	θ_g	30.0	20.0	mV
τ_r^1	17.5	–	ms	σ_m	15.0	10.0	mV
τ_h^0	1.0	0.05	ms	σ_h	-3.1	-12.0	mV
τ_n^0	1.0	0.05	ms	σ_n	8.0	14.0	mV
τ_r^0	40.0	–	ms	σ_r	-2.0	-2.0	mV
ϕ_h	5	0.1		σ_a	7.8	2.0	mV
ϕ_n	5	0.3		σ_b	-0.1	–	
ϕ_r	2	1.0		σ_s	8.0	2.0	mV
k_1	15.0	30.0		σ_h^τ	-3.0	-12.0	mV
$k_{\rm Ca}$	22.5	3.0		σ_n^τ	-26.0	-12.0	mV
σ_r^τ	-2.2	–	mV	σ_g^H	8.0	2.0	mV
α	5.0	2.0	ms^{-1}	β	1.0	0.045	ms^{-1}
$I_{{\rm app},j}$	$\mathcal{N}(10, 0.015^2)$	-4.24	pA/μm^2	τ_r	–	30	ms
C_m	1.0	1.0	pF/μm^2	ε_j	$5 \cdot 10^{-5}$	$\mathcal{N}(0.0055, [2 \cdot 10^{-5}]^2)$	ms^{-1}

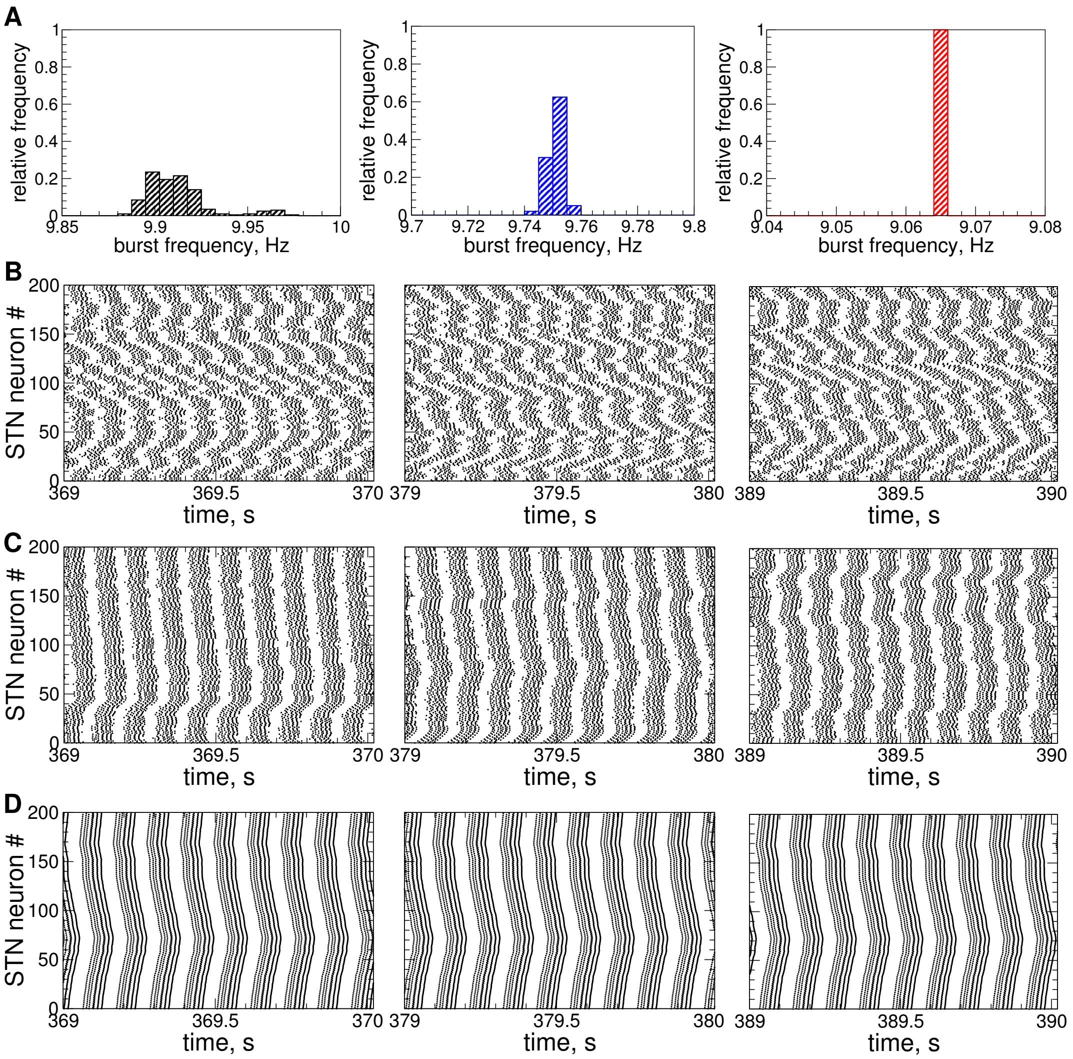

Figure S1. Collective dynamics of STN-GPe neurons (1) - (4) without stimulation. (A) Distribution histograms of the individual average bursting frequencies (number of bursts per second) of the stimulation-free STN neurons for coupling parameter $g_{G\to S} = 1.28$ nS/μm^2 (left plot), $g_{G\to S} = 1.38$ nS/μm^2 (middle plot) and $g_{G\to S} = 1.7$ nS/μm^2 (right plot). (B) - (D) The corresponding spike raster plots of $N = 200$ STN neurons at different time instances, where the spike onsets are indicated by black dots for (B) $g_{G\to S} = 1.28$ nS/μm^2, (C) $g_{G\to S} = 1.38$ nS/μm^2 and (D) $g_{G\to S} = 1.7$ nS/μm^2.

REFERENCES

Benabid, A. L., Chabardes, S., Mitrofanis, J., and Pollak, P. (2009). Deep brain stimulation of the subthalamic nucleus for the treatment of Parkinson's diseaseegge. *Lancet Neurol.* 8, 67–81. doi:10.1016/S1474-4422(08)70291-6

Hammond, C., Bergman, H., and Brown, P. (2007). Pathological synchronization in Parkinson's disease: networks, models and treatments. *Trends Neurosci.* 30, 357–364. doi:10.1016/j.tins.2007.05.004. July INMED TINS special issue – Physiogenic and pathogenic oscillations: the beauty and the beast

Park, C., Worth, R. M., and Rubchinsky, L. L. (2011). Neural dynamics in parkinsonian brain: The boundary between synchronized and nonsynchronized dynamics. *Phys. Rev. E* 83, 042901. doi:10.1103/PhysRevE.83.042901

Rubin, J. E. and Terman, D. (2004). High frequency stimulation of the subthalamic nucleus eliminates pathological thalamic rhythmicity in a computational model. *J. Comput. Neurosci.* 16, 211–235

Terman, D., Rubin, J. E., Yew, A. C., and Wilson, C. J. (2002). Activity patterns in a model for the subthalamopallidal network of the basal ganglia. *J. Neurosci.* 22, 2963–2976. doi:20026266
